# Single-cell RNA-seq of esophageal squamous cell carcinoma cell line with fractionated irradiation reveals radioresistant gene expression patterns

**DOI:** 10.1186/s12864-019-5970-0

**Published:** 2019-07-25

**Authors:** Ling Yang, Xiaoyan Zhang, Qiang Hou, Ming Huang, Hongfang Zhang, Zhenzhen Jiang, Jing Yue, Shixiu Wu

**Affiliations:** 1 Hangzhou Cancer Institute, Hangzhou Cancer Hospital, Hangzhou, Zhejiang Province 310002 People’s Republic of China; 2 National Cancer Center/National Clinical Research Center for Cancer/Cancer Hospital & Shenzhen Hospital, Chinese Academy of Medical Sciences and Peking Union Medical College, No.113 Baohe Street Longgang District, Shenzhen, China

**Keywords:** Single-cell RNA-seq, Transcriptome, Esophageal squamous cell carcinoma, Radioresistance, Gene expression

## Abstract

**Background:**

Esophageal squamous cell carcinoma (ESCC) cells are heterogeneous, easily develop radioresistance, and recur. Single-cell RNA-seq (scRNA-seq) is a next-generation sequencing method that can delineate diverse gene expression profiles of individual cells and mining their heterogeneous behaviors in response to irradiation. Our aim was using scRNA-seq to describe the difference between parental cells and cells that acquired radioresistance, and to investigate the dynamic changes of the transcriptome of cells in response to FIR.

**Results:**

We sequenced ESCC cell lines KYSE180 with and without fractionated irradiation (FIR). A total of 218 scRNA-seq libraries were obtained from 88 cells exposed to 12 Gy (KYSE-180-12 Gy), 89 exposed to 30 Gy (KYSE-180-30 Gy), and 41 parental KYSE-180 cells not exposed to FIR. Dynamic gene expression patterns were determined by comprehensive consideration of genes and pathways. Biological experiments showed that KYSE-180 cells became radioresistant after FIR. PCA analysis of scRNA-seq data showed KYSE-180, KYSE-180-12 Gy and KYSE-180-30 Gy cells were discrete away from each other. Two sub-populations found in KYSE-180-12 Gy and only one remained in KYSE-180-30 Gy. This sub-population genes exposure to FIR through 12 Gy to 30 Gy were relevant to the PI3K-AKT pathway, pathways evading apoptosis, tumor cell migration, metastasis, or invasion pathways, and cell differentiation and proliferation pathways. We validated DEGs, such as *CFLAR*, *LAMA5*, *ITGA6*, *ITGB4*, and *SDC4* genes, in these five pathways as radioresistant genes in bulk cell RNA-seq data from ESCC tissue of a ESCC patient treated with radiotherapy and from KYSE-150 cell lines.

**Conclusions:**

Our results delineated the divergent gene expression patterns of individual ESCC cells exposure to FIR, and displayed genes and pathways related to development of radioresistance.

**Electronic supplementary material:**

The online version of this article (10.1186/s12864-019-5970-0) contains supplementary material, which is available to authorized users.

## Background

Esophageal squamous cell carcinoma (ESCC) is a common subtype of esophageal carcinoma, especially in China [1]. Radiotherapy is the choice of treatment for locally advanced ESCC, but the prognosis is dismal due to development of radioresistance. Several studies showed that insulin-like growth factor 2 mRNA-binding protein 3 was identified as a radioresistance factor in ESCC [2]; miR-205 promoted radioresistance of ESCC by enhancing DNA repair, inhibiting apoptosis and activating epithelial-mesenchymal transition [3]; the eEF2K could induce progression and radioresistance in ESCC [4]. Numerous genetic alterations, including the activation of oncogenes and the inactivation of tumor-suppressor genes, accumulate during the development of ESCC [5], and high rates of local-recurrence and acquired radioresistance are closely related to the high heterogeneity of ESCC cell populations. Therefore, obtaining a deeper knowledge of the heterogeneous behaviors of ESCC cells and then designing strategies that increase the radio-sensitivity of ESCC or reverse the resistance to irradiation are key issues for both researchers and clinicians [6].

Advances in next-generation sequencing (NGS) technology have enabled genome, transcriptome and methylome analyses [7] and several NGS-based genome and transcriptome analysis of ESCC have been recently reported [1, 5, 8–12]. However, the number of cells that are usually required for conventional NGS-based analysis result in provision of an averaged message that misses some critical subpopulation information [13]. Single-cell RNA sequencing (scRNA-seq) is a method of whole transcriptome amplification, library construction and NGS that is suitable for analysis of single cancer-cells heterogeneity, especially for investigating dynamic changes of gene expression pattern in cell populations exposed to irradiation. Previously, we performed bulk-cell RNA-seq and scRNA-seq on ESCC cell line KYSE-30 cells with and without induced paclitaxel resistance, and revealed molecular signals, especially induced proteasomes and deduced HIF-1 signaling, to intrinsic and acquired paclitaxel resistance in ESCC cells [14]. Herein, we performed scRNA-seq to describe the heterogeneity of gene expression in KYSE-180 cells with and without exposure to fractionated irradiation (FIR), and to investigate the dynamic changes of the transcriptome of cells in response to FIR. Our aim was to describe the difference between parental cells and cells that acquired radioresistance. scRNA-seq data obtained from KYSE-180 cells before exposure to FIR served as a control, and post-FIR data was obtained after cumulative doses of 12 Gy and 30 Gy. Bioinformatic analysis revealed distinct transcriptome features that corresponded to the different FIR treatments. Furthermore, two sub-clones were found in the 12 Gy cell population and only one sub-clone survived in 30 Gy cells, and five radiation-resistant pathways were deduced. Twelve radioresistant and up- or down-regulated genes validated in ESCC tissue samples from ESCC patient with radiotherapy and KYSE-150 cells that with the same irradiation-treatment. Overall, the objective was to provide new data on radioresistance in ESCC based on scRNA-seq analysis.

## Results and discussion

### KYSE-180 cells resist FIR in vitro

**Fig. 1:**
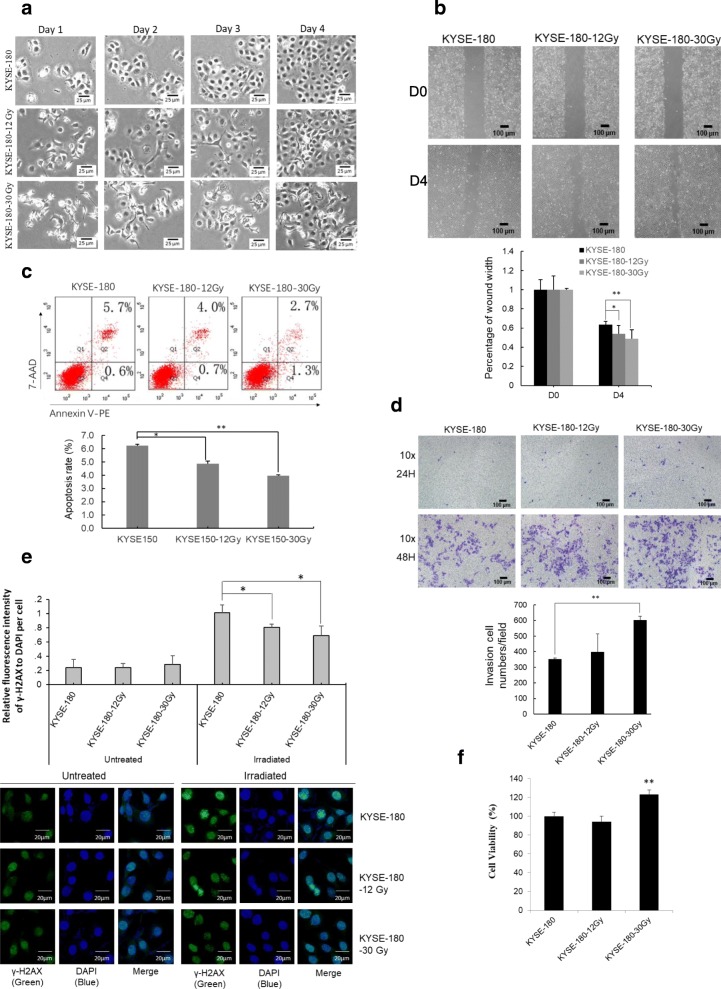
KYSE-180 cells acquired radioresistance in vitro. **a**. Morphological image of KYSE-180, KYSE-180-12 Gy and KYSE-180-30 Gy. **b**. Wound healing assay showed migration of KYSE-180, KYSE-180-12 Gy and KYSE-180-30 Gy. D0, Day 0; D4: Day 4. **c**. FACS analysis with Annexin V-PE and 7-AAD showing apoptosis results of KYSE-180, KYSE-180-12 Gy and KYSE-180-30 Gy. **d**. Transwell assay showed invasion of KYSE-180, KYSE-180-12 Gy and KYSE-180-30 Gy. **e**. Immunofluorescence analysis of γ-H2AX expression 2 h after 4 Gy of FIR in control KYSE-180 cells and in FIR-treated KYSE-180-12 Gy and KYSE-180-30 Gy cells. Green: γ-H2AX; Blue: DAPI. **f**. Surviving KYSE-180 cells with and without FIR exposure identified by CCK-8 assay. * means *P* < 0.05; ** represents *P <* 0.01

The in vitro model used to evaluate dynamic changes of KYSE-180 cells in response to FIR was performed: A 30 Gy cumulative irradiation dose (2 Gy/day for 3 days with 4 days to recover) required five cycles (the corresponding cells named KYSE-180-30 Gy), and a 12 Gy cumulative dose needed 2 cycles (the corresponding cells named KYSE-180-12 Gy). The model was a simulation of radiotherapy routine applied in ESCC patients as reported [15, 16]. With day-to-day irradiation, the cell morphology (from cuboidal to an elongated spindle shape) (Fig. 1a) and the abilities of migration (Fig. 1b) and evading apoptosis (Fig. 1c) showed obvious changes after receiving a 12 Gy of cumulative dose, and the abilities of invasion significant changes after 30 Gy cumulative irradiation dose (Fig. 1d). Therefore, we determined three sampling points: KYSE-180-0Gy, KYSE-180-12 Gy and KYSE-180-30 Gy.

To determine whether KYSE-180 cells became radioresistant after FIR, we measured the expression of γ-H2AX, a marker of irradiation-induced double-strand breaks [17, 18], in KYSE-180 cells with and without FIR exposure. The results showed that γ-H2AX expression in both KYSE-180-12 Gy and KYSE-180-30 Gy cells was significantly lower than it was in KYSE-180 control cells 2 h after 4 Gy of radiation (Fig. 1e). In addition, the cell counting kit-8 (CCK-8) assay found that proliferation of KYSE-180-30 Gy cells was significantly higher than proliferation of parental KYSE-180 cells (Fig. 1f). Overall, these results confirm that KYSE-180 cells become radioresistant after FIR, especially after a cumulative 30 Gy of irradiation dose.

### scRNA-seq of KYSE-180 in response to FIR

To analyze the divergence of gene expression in response to FIR and the dynamic changes in differentially expressed genes (DEGs) of KYSE-180 cells with two different cumulative doses of FIR (12 Gy and 30 Gy), we constructed a series of scRNA-seq libraries from KYSE-180 cells using smart-seq 2. We then sequenced 229 FIR-treated cells (0 Gy, 12 Gy, or 30 Gy) after 3 days of recovery about 2 million high quality reads for each cell. RNA-seq reads were mapped to the reference human genome, with an average of 65.4% reads mapped within the genome. To ensure the fidelity of the data, we discarded cells with fewer than 1800 detected genes. We discarded genes with less than ten counts in each cell, and kept genes that were seen in at least five cells. To test the reproducibility of data, we repeated sequencing using the same templates, and the high correlation (*r* = 0.96) that we obtained confirmed the high reliability of the sequencing data (Additional file 1: Figure S1a). To obtain accurate estimates of expression level, we normalized the sequencing depth using the Bioconductor DEseq2 package to ensure that all cells had approximately the same median read depth (Additional file 1: Figure S1b). A total of 218 cells passed this filter, leaving 41 KYSE-180 cells, 88 of KYSE-180-12 Gy cells, and 89 cells KYSE-180-30 Gy cells. Overall, the results suggested that our scRNA-seq data was of sufficient quality for further analysis.

To examine gene expression levels of individual cells, we used the relative standard deviations (RSDs) of normalized gene counts to estimate the dispersion of expression levels. Firstly, genes with higher expression levels had lower degrees of dispersion (Additional file 1: Figure S1c). It thus appeared that genes with lower normalized counts had higher relative dispersions, and genes with log ratios of normalized counts > 4 (> 2 log_2_ values) would have a depth sufficient to represent their true expression status. To exclude technical errors, we estimated the reproducibility of independent experiments (*r* = 0.98; Additional file 1: Figure S1d). We then calculated the RSD for each gene in all cells, and selected 1000 genes with minimum RSD values for Kyoto Encyclopedia of Genes and Genomes (KEGG) pathway enrichment analysis, which could explore some relatively stable pathways under radiation stress. Twenty-seven pathways were detected, including ribosome (KEGG ID: hsa03010), spliceosome (KEGG ID: hsa03040) and Proteasome (KEGG ID: hsa03050) (Additional file 2: Table S1). These results indicated that some important pathways related protein processing had less variation in response to irradiation.

**Fig. 2:**
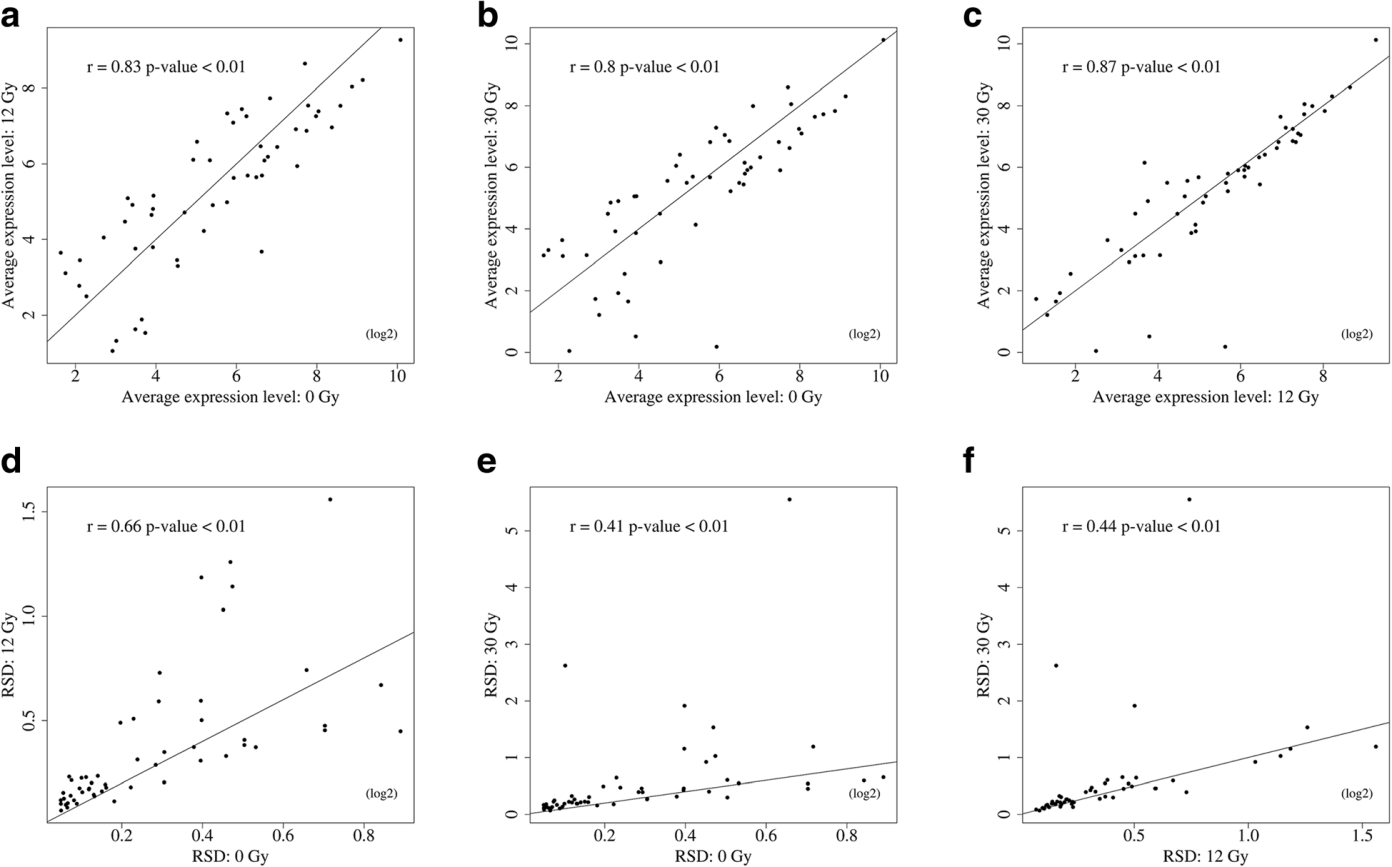
Basic gene expression statistics. Comparison of average expression (**a**-**c**) and RSD (**d**-**f**) of cancer-related genes among the experimental radiation groups. The two figures in the middle column show the lowest difference of average expression (**b**) and RSD (**e**) between control and 30 Gy cells, implying that the expression levels and heterogeneity of cancer-related genes changed with radiation dose. Pearson correlation coefficient was used

To examine the divergence of response to irradiation, we compared the expression levels of cancer-related genes [19] (Additional file 2: Table S1) after receiving different radiation doses (Fig. 2). Although there is no statistical difference, the correlation coefficient of average expression level of cancer-related genes between 30 Gy and 0 Gy (*r* = 0.8, Fig. 2b) was slightly lower than the coefficient between 12 Gy and 0 Gy (*r* = 0.83, Fig. 2a). In contrast, the high correlation between 12 Gy and 30 Gy also showed the difference of cancer-related genes was small (*r* = 0.87, Fig. 2c). From Fig. 2d to Fig. 2f, we used RSD to explore the status of highly variable genes. Relative to 0 Gy, 30 Gy induced higher variability of genes. The expression of most cancer related genes showed high correlation among these three groups. However, several genes showed exclusive expression pattern across control and two FIR groups.

### Two subpopulations existent in 12 Gy cells and only one cluster in 30 Gy cells

**Fig. 3:**
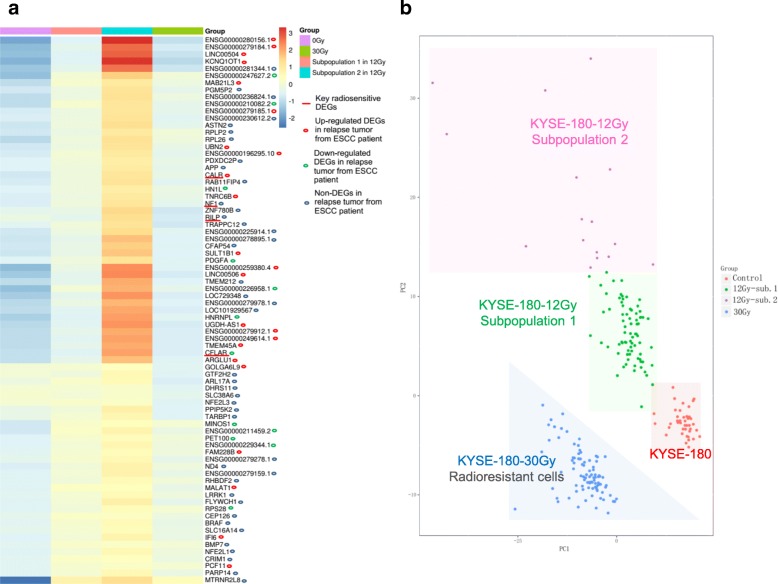
DEGs (**a**) in two subpopulations of 12 Gy cells and PCA (**b**) of DEGs shows three radiation groups with obviously different distributions. a. The DEGs in the two subpopulations of 12 Gy in all cell groups suggested that subpopulation 1 was similar to the 30 Gy cells, and increased expression of those genes led to killing by radiation. b. The 30 Gy group was slightly more discrete than the control population and the difference between the 12 Gy group and control population was greater

To gain insight into the molecular characteristics of KYSE-180 parental cells and irradiation cells, we compared the DEGs in 41 KYSE-180, 88 KYSE-180-12 Gy, and 89 KYSE-180-30 Gy cells by ANOVA at cutoff difference of 1.5-fold (KYSE-180-12 Gy vs. KYSE-180 > 1.5-fold, or KYSE-180-30 Gy vs. KYSE-180 > 1.5-fold; *P* < 0.05) (Additional file 2: Table S2). A total of 786 DEGs were identified, 284 DEGs that were radiation-induced (Additional file 1: Figure S2, 126 and 158 DEGs in patterns 1 and 3, respectively; Additional file 2: Table S2) and 502 DEGs that were radiation-reduced (Additional file 1: Figure S2, 101 and 401 DEGs in patterns 2 and 4, respectively; Additional file 2: Table S2). Compared with parental cells, The DEGs in pattern 1 identified two subpopulations in KYSE-180-12 Gy cells (Additional file 1: Figure S2; 12 Gy-sub.1 and 12 Gy-sub. 2). The expression levels of these DEGs induced more in subpopulation 2 of KYSE-180-12 Gy (Additional file 1: Figure S2; 12 Gy-sub. 2), and the key DEGs specifically involved in forming this subtype were obviously up regulated (Fig. 3a with four cell outliers; Additional file 2: Table S3). The DEGs in pattern 2 were significantly downregulated in KYSE-180-30 Gy, while no obvious change observed in KYSE-180-12 Gy cells. Based on all DEGs (Pattern 1–4), we found the expression patterns of DEGs in subpopulation 1 of KYSE-180-12 Gy were similar to those in KYSE-180-30 Gy (Additional file 1: Figure S2). We then evaluated principal component analysis (PCA) in 218 individual cells from the three sets of samples selected by the expression level of all DEGs (Fig. 3b). Individual cells in each set of samples formed distinct clusters. KYSE-180-12 Gy and KYSE-180-30 Gy clustered away from parental cells and subpopulation 2 of KYSE-180-12 Gy cells deviated from the center of their cluster (Fig. 3b). The position of cell groups might show different cell features of RNA expression, indicating different cell types or cell fates.

In the disease ontology analysis, many DEGs, including *CFLAR*, *BRAF*, *CD24*, *BMP7*, *LAMB3*, *ITGA6*, *ITGB4*, *S100A9*, *AKT3*, relate to squamous cell carcinoma and esophageal cancer (Additional file 2: Table S4). The KEGG analysis (Additional file 2: Table S5) found that a number of DEGs were related to pathways in cancer (KEGG ID: hsa05200), including six upregulated DEGs (*BRAF*, *LAMA5*, *NFKBIA*, *ITGA6*, *LAMB3*, and *LAMB2*) and two downregulated DEGs (*CYCS*, *AKT3*). *BRAF* and *RHEB* also participate in the mTOR signaling pathway; *CFLAR*, *NFKBIA*, and *AKT3* participated in an apoptosis pathway (KEGG ID: hsa04210). The enrichment GO analysis of different gene patterns (Additional file 2: Table S8) showed that some genes of pattern 1 belonging to nuclear-transcribed mRNA catabolic process, some genes of pattern 2 involved in some progresses of antigen processing and presentation, many genes of pattern 3 and pattern 4 were associated in cell structure and metabolism.

### Genes and five pathways related to development of radioresistance in KYSE-180-12 Gy and − 30 Gy cells

Pattern 2 DEGs in Fig. 3 were significantly down regulate in KYSE-180-30 Gy. These DEGs were closely related with radioresistant. We found 10 genes in metabolic pathways in this pattern were all down regulated, indicated decreased metabolism in radioresistant cells after 30 Gy of irradiation (Based on DAVID analysis; Additional file 2: Table S2, pattern 3). When the DEGs in Pattern 2 and Pattern 4 combined, 66 DEGs in metabolic pathways and 10 DEGs in cell cycle were reduced.

**Fig. 4:**
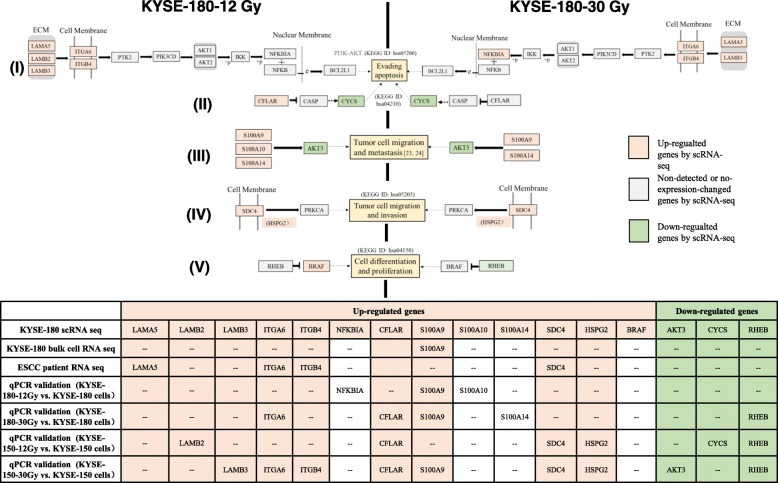
Dynamic changes of five radioresistant pathways in KYSE-180-12 Gy and − 30 Gy cells. KEGG ID: hsa05200 (I), hsa04210 (II), hsa05205 (IV), and hsa04150 (V) should be induced in both two doses of FIR, and are related to avoiding apoptosis and promoting tumor cell migration, invasion, differentiation, and proliferation. The (III) pathway was deduced from previously published data [20, 21]. The validation results from bulk cell data are shown in the lower table

Based on the disease ontology and KEGG results, we traced out five radioresistant pathways (Fig. 4) that might be relate to avoiding apoptosis, promoting cell migration, or increasing proliferation of KYSE-180 after exposure to a cumulative irradiation. These five pathways characterized by dynamic changes after cumulative irradiation with 12 Gy and 30 Gy (Fig. 4), and these pathways were matched with the cellular phenotypic changes in Fig. 1. Firstly, the PI3K–AKT signaling pathway (KEGG ID: hsa05200) was activated, with some DEGs (*LAMA5*, *LAMB2*, *LAMB3*, *ITGA6*, and *ITGB4*) upregulated at both 12 Gy and 30 Gy of FIR-doses, and with *NFKBIA* only upregulated at 30 Gy. The PI3K-AKT pathway may be a primary pathway to protect KYSE-180 from apoptosis after irradiation. Secondly, the *CYCS* gene-based apoptosis pathway (KEGG ID: hsa04210) was inhibited. Downregulation of *CYCS* induced after 12-Gy irradiation. Thirdly, an *S100AX*–*AKT3*-related pathway was activated, which increased migration and metastasis of KYSE-180-12 Gy and KYE-180-30 Gy cells. In line with this, a recent study reported that upregulating *S100A4* can downregulate *AKT3*, but not *AKT1* and *AKT2*, and increase migration and metastasis in triple negative breast cancer cells [20]. *S100A9* is a key mediator of tumor cell aggressiveness [21]. We found *S100A9* and its partner *S100A14* were upregulated at 12 Gy and 30 Gy, while *S100A10* was induced at 12 Gy, and *AKT3* significantly downregulated only at 12 Gy. Therefore, induction of *S100A9* or *S100A10* or *S100A14*, and downregulation of *AKT3* may increase migration and metastasis of KYSE-180 cells after irradiation. Fourthly, we found that *SDC4* and *HSPG2*, which are two proteoglycans in a cancer pathway (KEGG ID: hsa05205), were upregulated in both KYSE-180-12 Gy and − 30 Gy cells. The KEGG ID: hsa05205 pathway showed that induced expression of both genes related to the promotion of tumor cell migration and invasion. Fifthly, the mTOR signaling pathway, which relate to cell proliferation, can be activated because we found *RHEB* and *BRAF* induced after 12 Gy and reduced after 30 Gy of irradiation, respectively. BRAF is a member of a family of serine-threonine protein kinases, including RAF1, BRAF and ARAF, which can phosphorylate and activate MKK1/2; BRAF has relatively greater ability to catalyze this reaction than the other kinases [22]. A previous report showed that exposure to doses of less than 2 Gy will activate *RAF1*, but not *BRAF*, and then induce proliferation of A431 squamous carcinoma cells [23]. Our scRNA-seq results revealed that KYSE-180 ESCC cells can activate *BRAF*, but not *RAF1*, and can reduce *RHEB*, which inhibits *BRAF* (KEGG ID: hsa04150), after irradiation. This might account for the radiation-induced proliferation of KYSE-180-30 Gy cells. Moreover, the inhibition of *RHEB* in *BRAF* (KEGG ID: hsa04150) might enhance the apoptotic effects induced by radiation [24]. *BRAF* is also associated with cell proliferation, the downregulation of *RHEB* and upregulation of *BRAF* can avoid apoptosis and increase proliferation.

### Validation study in ESCC cell lines and an ESCC patient

We obtained bulk cell RNA-seq data from KYSE-180, KYSE-180-12 Gy, KYSE-180-30 Gy cells, and primary and recurrent tumor tissues from an ESCC radiotherapy patient. The data were analyzed to validate the single-cell results and to determine the correlations between the DEGs found in scRNA-seq data of KYSE-180 with and without FIR (Fig. 4 and Additional file 2: Table S6). There was a high level of concordance of the differential expression measurements of data at *S100A9* (the same expression pattern of this gene in bulk cell RNA-seq data of KYSE-180), *LAMA5*, *ITGA6*, *ITGB4*, *SDC4* (the same expression pattern of this gene in tissue RNA-seq data from ESCC patient) (Fig. 4). Interestingly, we did not find different expression patterns of *LAMB2*, *LAMB3*, *HSPG2*, *S100A10*, *S100A14*, *NFKBIA*, or *AKT3* in the bulk cell or tissue RNA-seq data that might be advantage of single-cell RNA-seq.

In order to validate RNA-seq data of KYSE-180, we checked all DEGs of five key radioresistant-related pathways (Fig. 4, I-V) in KYSE-180 cells again and in KYSE-150 cells by using qPCR (table in Fig. 4, Additional file 1: Figures S3 and S4). Finally, we found that several DEGs in each radioresistant-related pathway were in concordance with KYSE-180, ESCC patient samples and KYSE-150 cells: *LAMA5*, *LAMB2*, *LAMB3*, *ITGA6* and *ITGB4* in pathway I, *CFLAR* and *CYCS* in pathway II, *S100A9* and *AKT3* in pathway III, *SDC4* and *HSPG2* in pathway IV, *RHEB* in pathway V (table in Fig. 4, marked with yellow for up-regulated genes and green for down-regulated genes). We further analyses the corresponding cellular phenotypic changes of KYSE-150 cells after 12 Gy and 30 Gy treatment, we found the abilities of invasion (Additional file 1: Figure S5a), evading apoptosis (Additional file 1: Figure S5b) and proliferation (Additional file 1: Figure S5c) of KYSE-150 showed significant changes after receiving a 12 Gy or 30 Gy cumulative dose. These results from mRNA and phenotype levels indicated the DEGs up-regulated or down-regulated in five radioresistant-related pathways were critical for development of radioresistance in ESCC.

## Conclusions

We analyzed how the diversity of individual KYSE-180 cells changed in response to an FIR stimulus of 12 Gy and 30 Gy cumulative radiation doses. We investigated how heterogeneity is regulated using individual transcriptome profiles. As far as we know, this is the first study that has described the detailed heterogeneity of ESCC cells after irradiation at the single-cell-transcriptome level. Our study revealed that gene expression of single cells was highly varied and was characteristic of particular genes and pathways related to the cumulative dose of FIR. Moreover, we observed that some KYSE-180-12 Gy cells deviated from the center of their cluster such that heterogeneity was more pronounced among KYSE-180-12 Gy cells than in the parental KYSE-180 or KYSE-180-30 Gy cells. A wide variety of DEGs were included and diverse clusters were observed after irradiation, which likely reflects the impact of FIR on various biological processes and signaling pathways in KYSE-180 cells.

We found two single-cell KYSE-180-12 Gy subpopulations and no subpopulation in KYSE-180-30 Gy (Additional file 1: Figure S2). Although there were some differences between KYSE-180-12 Gy and KYSE-180-30 Gy of DEGs, a similar expression pattern of molecular signatures was observed between the subpopulation 2 of KYSE-180-12 Gy and KYSE-180-30 Gy (Fig. 3a). The clustering algorithm was described in the section of Computational Procedures. KYSE-180-30 Gy cells derived from a subpopulation (most likely subpopulation 1) of KYSE-180-12 Gy cells. Their expression patterns may have originally been more homogeneous than their parental KYSE-180 cells. However, the significant difference of DEGs in pattern 2 of KYSE-180-12 Gy cells and in KYSE-180-30 Gy cells is an excellent example of the dynamic changes of KYSE-180 cells in response to different cumulative doses of irradiation. Based on these results, we deduced that the homogeneous population of KYSE-180 cells became heterogeneous after receiving 12 Gy cumulative irradiation.

The analysis of DEG patterns revealed five important radioresistant pathways. The similar gene expression patterns of DEGs in these five KYSE-180-12 Gy and KYSE-180-30 Gy pathways cross validated the results, showed the importance of these pathways in ESCC cells radioresistance, and to some extent validated the accuracy of the scRNA-seq data.

Our analysis provides inferential evidence for dynamic transitions of ESCC cells in response to FIR-treatment. A better understanding of such changes in cellular states in ESCC is crucial for establishing representative experimental models and developing radiotherapy strategies.

## Methods

### Cell culture and irradiation treatment

The human ESCC cell line KYSE-180 (Catalog: ZH1036) was purchased from Shanghai Zhonghua Biotechnological Co. LTD, and KYSE-150 (Catalog: TCHu236) was purchased from the Cell Bank of the Chinese Academy of Sciences, Shanghai, China, and they were both passaged for less than 4 months. The cell line was authenticated by short tandem repeat analysis. The cells were cultured in RPMI-1640 (Gibco, Thermo Fisher Scientific, Waltham, MA, USA) with 500 ng/mL penicillin-streptomycin and 10% fetal bovine serum (FBS), and incubated at 37 °C in 5% CO_2_/95% air.

For irradiation, (3 × 10^6^) cells were plated in 75 cm^2^ culture flasks. When cells reached approximately 70% confluence, the medium was renewed, and the culture was irradiated with 2 Gy X-rays using a linear accelerator (Elekta, Stockholm, Sweden) at an average dose rate of 100 cGy per minute, a 20 × 20 cm field, and source-skin distance of 100 cm. Immediately after irradiation, cells were returned to the incubator. The cultures were irradiated with 2 Gy again on days 2 and 3 and then maintained in culture for the next four days to recover. When 90% confluence was reached, cells were trypsinized and subcultured into new flasks. These procedures were repeated either two or five times to achieve a total dose of either 12 Gy (KYSE-180-12 Gy and KYSE-150-12 Gy) or 30 Gy (KYSE-180-30 Gy and KYSE-150-30 Gy). Parental cells used the radiation control were trypsinized, counted, and passaged under the same conditions without irradiation. When the repeated procedures had been completed, cells were trypsinized and washed, and single cells captured by micromanipulator for scRNA-seq. The remaining cells were collected for bulk cell RNA-seq.

### Cell matrigel assay

Cells at a density of 2 × 10^4^ cells/100ul RPMI-1640 were seeded in the upper wells of Matrigel-precoated transwell plates (Corning Costar Co., Lowell, CA, USA). 500ul RPMI-1640 containing 10% FBS and 500 ng/mL penicillin-streptomycin were added in the lower chambers. At 24 h or 48 h, the non-invaded cells were wiped out, and the membranes were treated with 4% formaldehyde 15 min at room temperature for fixation. Then the membranes were stained by crystal violet staining. Ten random fields were counted per chamber by using Olympus inverted microscope (Olympus, Tokyo, Japan).

### Wound healing assay

Cells were seeded in 6-well culture plates at a density of 3 × 10^5^ cells/well and cultured to 90% confluence. Cellular monolayer was wounded with a sterile 100-ul pipette tip and washed by PBS, then added with RPMI-1640. The image were captured at every 24 h using a digital camera until the wound closed.

### Apoptosis

Apoptosis was determined by using the Annexin V-PE and 7-AAD apoptosis detection kit (Becton-Dickinson, 559763, USA). Cells were seeded in 6-well plate at a density of 3 × 10^5^ cells/well and cultured. After 48 h, supernatant and trypsin-digested cells were all collected and washed twice with PBS. Cells were resuspended in 1 × binding buffer at a concentration of 1 × 10^6^ cells/mL. Incubated the cell suspension with 5 ul Annexin V-PE and 5 ul of 7-AAD for 15 min at room temperature and protect from light. Analysis was carried out by flow cytometry (FACS Aria III, Becton-Dickinson, USA).

### Cell proliferation assay (CCK-8)

Cell proliferation was determined by CCK-8 assay (Dojindo Laboratories, Tokyo, Japan). Briefly, cells (5000 cells per well) were incubated 24 h or 48 h in 96-well plates, nonadherent cells were removed, and absorbance was measured at 450 nm with a SpectraMax i3 platform (Molecular Devices, Sunnyvale, California, USA). The proliferation of irradiated cells was expressed as a percentage normalized against untreated cell controls.

### Immunofluorescence assay of γ-H2AX expression

Adherent cells growing in 96-well plates were exposed to 4 Gy of radiation, and 2 h later, were fixed with acetone/methanol (1:1), and permeabilized with Triton-X 100 (0.1%) in phosphate buffered saline (PBS). Non-specific binding was blocked by 3% BSA in PBS. Cells were then incubated with anti-γ-H2AX antibody (Cell Signaling Technology, Beverly, MA, USA) for 2 h in PBS with 0.1% BSA followed by Alexa Fluor 488-conjugated secondary antibodies (Invitrogen, Waltham, MA, USA) to complete the indirect immunofluorescence procedure. Immunofluorescence images were obtained by confocal laser scanning microscopy.

### Single-cell RNA-seq of KYSE-180 cells

A series of single-cell RNA-seq libraries were prepared from KYSE-180, KYSE-180-12 Gy, and KYSE-180-30 Gy cells using smart-seq 2 methodology [25, 26] and a total of 229 cells were sequenced using the Illumina HiSeq 2500 high-throughput sequencing system. The scRNA-seq sequences were uploaded to the NCBI reference sequence database with the accession number GSE81812.

### KYSE-180 and ESCC patient bulk cell RNA-seq

Samples of ESCC primary and recurrent tumors obtained from an ESCC patient, and bulk KYSE-180, KYSE-180-12 Gy, and KYSE-180-30 Gy cells were collected. All patient sample collections, patient consent and recruitment followed institutional review board protocols from Hangzhou Cancer Hospital. The approval number is HZCH-2016-02. Patient in this study provided written informed consent for sample collection and data analyses. Total RNAs were extracted from these samples with an RNeasy Mini Kit (Qiagen, Valencia, CA, USA), and the quality of extracted RNA was evaluated with a Bioanalyzer 2100 (Agilent Technologies, Dublin, Ireland). Sequence libraries were prepared following the manufacturer’s instructions (TruSeq Stranded mRNA Library Prep Kit for NeoPrep), and then sequenced using an Illumina HiSeq 2500 system.

### Computational procedures

Original RNA-seq reads were trimmed by Trimmomatic, a read trimming tool for Illumina NGS data [27] and mapped to the GENCODE GRCh38 human reference genome (GENCODE v22) using Tophat 2 software [28]. The gene counts were obtained with the Rsubread [29] package, and cleaned by scde [30]. The expression levels were calculated by normalized gene counts and variance stabilizing transformation for clustering by the DESeq2 [31] package in the bioconductor. RSDs were calculated as standard deviations of each gene divided by average normalized gene counts [19]. To discover the intrinsic patterns of the KYSE-180 cell line, 1000 genes and their RSDs were selected for enrichment by KEGG pathways. Normalized counts of each gene were used for analysis of DEGs by Monocle 2 [32]. All cells were filtered following the default parameters in Monocle 2 package. No cell got mitochondria RNA more than 0.1%, so it suggested that no dead cells were considered in the downstream analysis. We used kmeans function in R language for clustering analysis of DEGs heatmap, and Sihouette method was used for K selection (Additional file 1: Figure S2). Based the results of K-means clustering for samples, we determined the sub-clones of cells in different groups. The Sincell package [33] was used to draw PCA plots, and the clusterProfiler [34] package was used for gene enrichment analysis.

### Real-time quantitative PCR (qPCR) validation of DEGs

DEGs in five radioresistant-related pathways were validated in KYSE-180 and KYSE-150 cells by qPCR. Briefly, 0 Gy, 12Gy and 30Gy-treated cells were collected and total RNA extracted. First-strand cDNA was synthesized by using PrimeScript RT Master Mix (Takara). qPCR involved use of the SYBR Premix EX Taq (Takara) on an ABI 7500 FAST real-time PCR system (Applied Biosystems, Foster City, CA, USA). The qPCR conditions were 95 °C for 30 s, followed by 40 cycles of 95 °C for 5 s and 60 °C for 30 s. The amplification efficiency of primers were examined firstly and the specificity of the primer amplicons was examined by melting curve analysis. The comparative Ct method was used for quantifying mRNA expression normalized to that of GAPDH (the internal control). qPCR primers used in this study were list in Additional file 2: Table S7.

### Statistics analysis

All experiments in this study were repeated independently three times, and the data were presented as means ±SD. The statistical analyses performed with two-tailed student’s t-test. *P* < 0.05 represents statistically significant.

## Additional files

Additional file 1: **Figure S1.** Quality control of basic gene expression statistics. a. Replicate sequencing showed high repeatability (*r* = 0.96) of expression level. b. The expression distribution after depth normalization revealed similar medians for all cells analyzed. c. The expression data of discrete genes suggested a correlation of increased expression with decreased discrete degree. Therefore, we could not include genes with a normalized count ratio of less than 2 log_2_. d. The RSDs of gene expression assayed by duplicate sequencing appear highly linear (*r* = 0.98). **Figure S2.** Heatmap of DEGs in the three treatment groups (0 Gy controls, 12 Gy, and 30 Gy). Four gene expression patterns are seen among all DEGs. Pattern 1 shows a slight upregulation after radiation, with two subpopulations of 12 Gy cells. Pattern 2 shows a strong downregulation after 30 Gy. Pattern 3 was similar to pattern 1, but no subpopulations were observed. Finally, pattern 4 shows many slightly downregulated genes. Four cells in the 12 Gy group had a completely different expression pattern, and were excluded as outliers after sequencing depth checking. **Figure S3.** qPCR validation of DEGs in five radioresistant-related pathways in Fig. 4 by new treated KYSE-180 cells, KYSE-180-12 Gy cells and KYSE-180-30 Gy cells. * means *P* < 0.05; ** represents *P <* 0.01. **Figure S4.** qPCR validation of DEGs in five radioresistant-related pathways in Fig. 4 by KYSE-150 cells, KYSE-150-12 Gy cells and KYSE-150-30 Gy cells. * means *P* < 0.05; ** represents *P <* 0.01. **Figure S5.** Radioresistance-associated cellular phenotypic evidences of KYSE-150 cells. a. Transwell assay showed invasion of KYSE-150, KYSE-150-12 Gy and KYSE-150-30 Gy. b. FACS analysis with Annexin V-PE and 7-AAD showing apoptosis results of KYSE-150, KYSE-150-12 Gy and KYSE-150-30 Gy. c. Surviving KYSE-150 cells with and without FIR exposure identified by CCK-8 assay. * means *P* < 0.05; ** represents *P* < 0.01. (PDF 28921 kb)

Additional file 2: **Table S1.** Basic pathways and Cancer-related genes. **Table S2.** Four patterns of DEGs. **Table S3.** Key DEGs of sub of KYSE-180 12Gy. **Table S4.** Disease ontology of 1.5 fold DEGs. **Table S5.** KEGG of DEGs-obtained. **Table S6.** Key DEGs of bulk cell. **Table S7.** qPCR primer sequences of mRNA. **Table S8.** The enrichment GO analysis of four patterns of DEGs. (XLS 302 kb)

## Data Availability

The scRNA-seq sequences were uploaded to the NCBI reference sequence database with the accession number GSE81812.

## Abbreviations

CCK-8: Cell counting kit-8
DEGs: Differentially expressed genes
ESCC: ESOPHAGEAL squamous cell carcinoma
FIR: Fractionated irradiation
KEGG: Kyoto Encyclopedia of Genes and Genomes
KYSE-180-12 Gy: KYSE-180 cells exposed to 12 Gy
KYSE-180-30 Gy: KYSE-180 cells exposed to 30 Gy
NGS: Next-generation sequencing
PCA: Principal component analysis (PCA)
RSD: Relative standard deviations
scRNA-seq: Single-cell RNA-seq
