# Supplementary material for: Single-cell RNA-seq of esophageal squamous cell carcinoma cell line with fractionated irradiation reveals radioresistant gene expression patterns
Source: BMC Genomics. 2019 Jul 25;20:611. doi: 10.1186/s12864-019-5970-0 (PMC6659267; doi:10.1186/s12864-019-5970-0)

Supplementary Figure 1

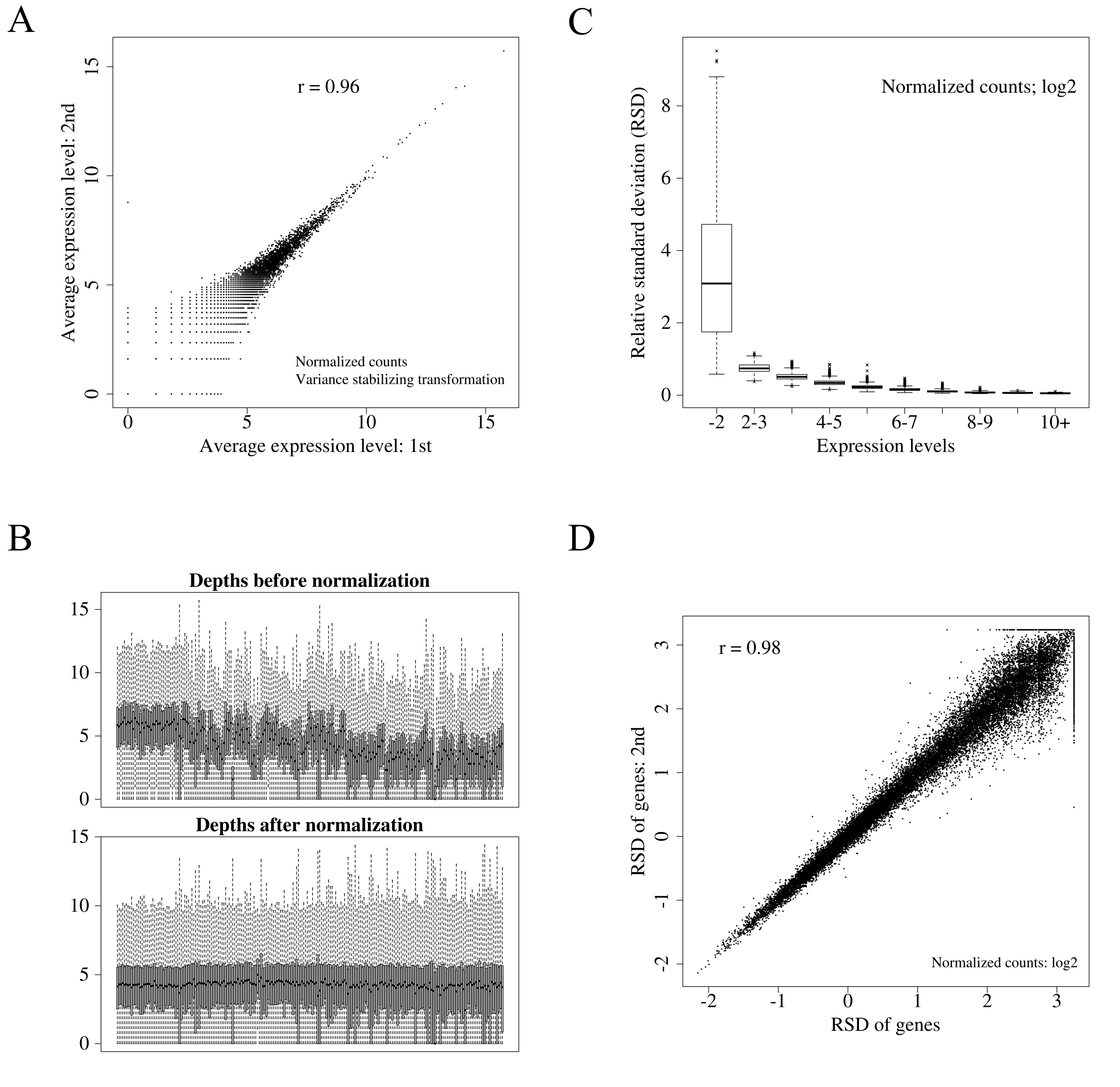

Supplementary Figure 2

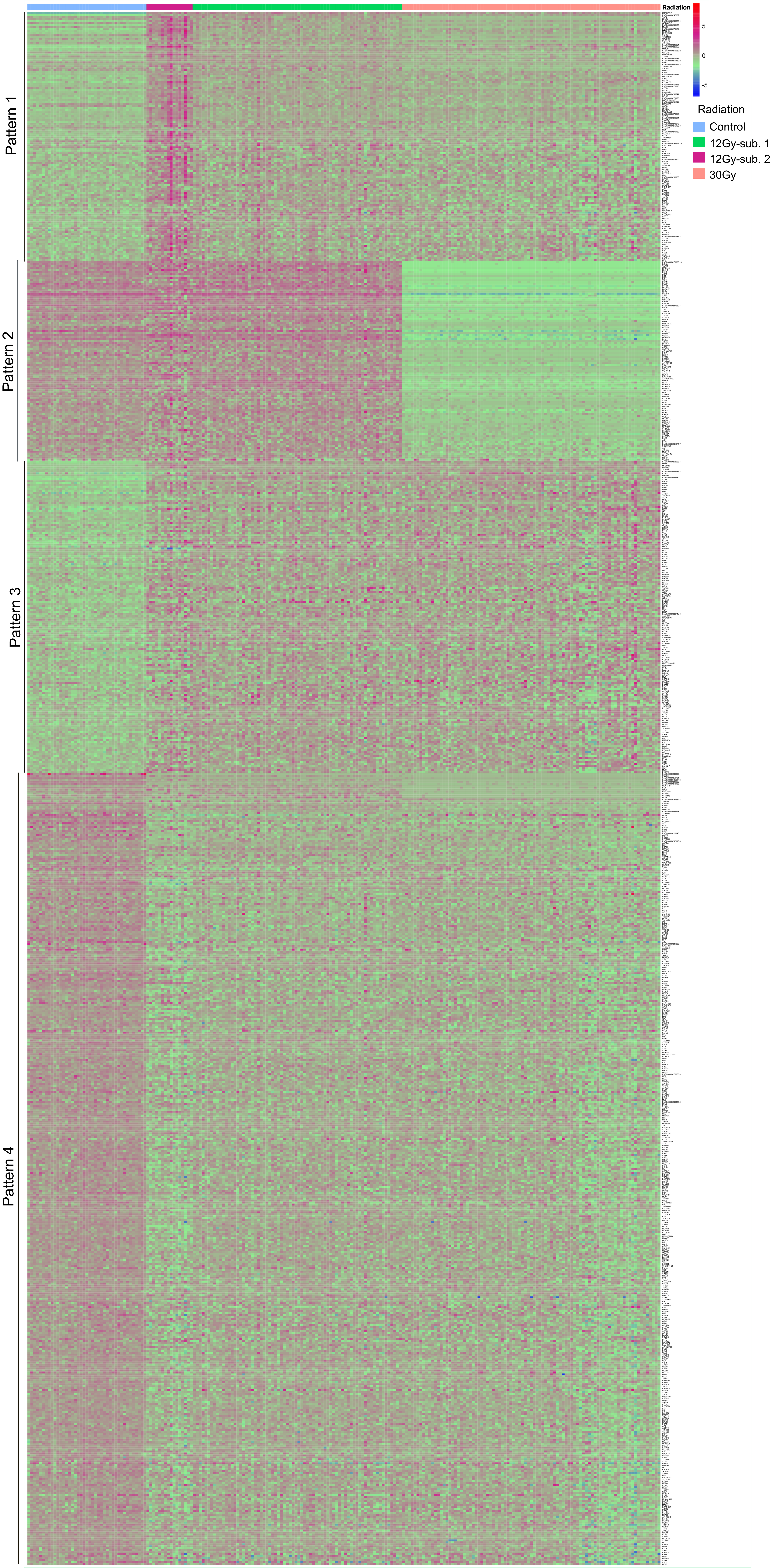

Supplementary Figure 3

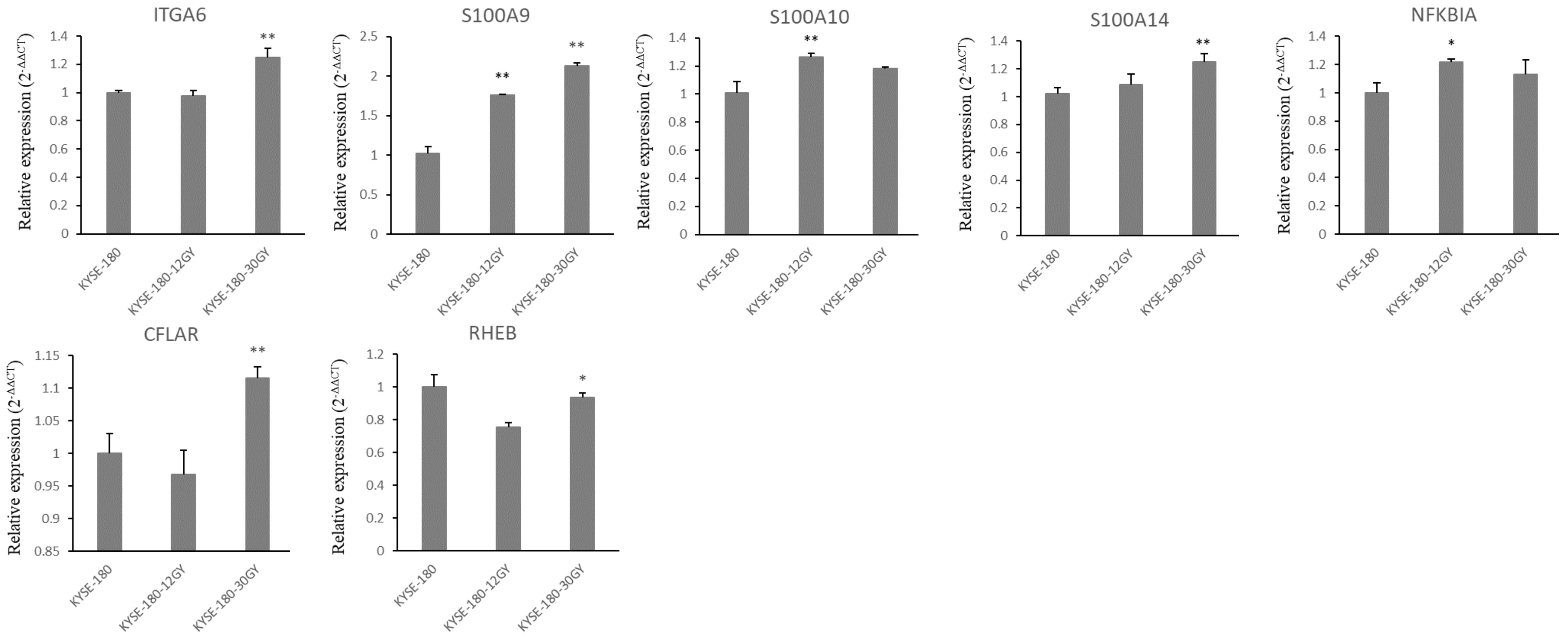

Supplementary Figure 4

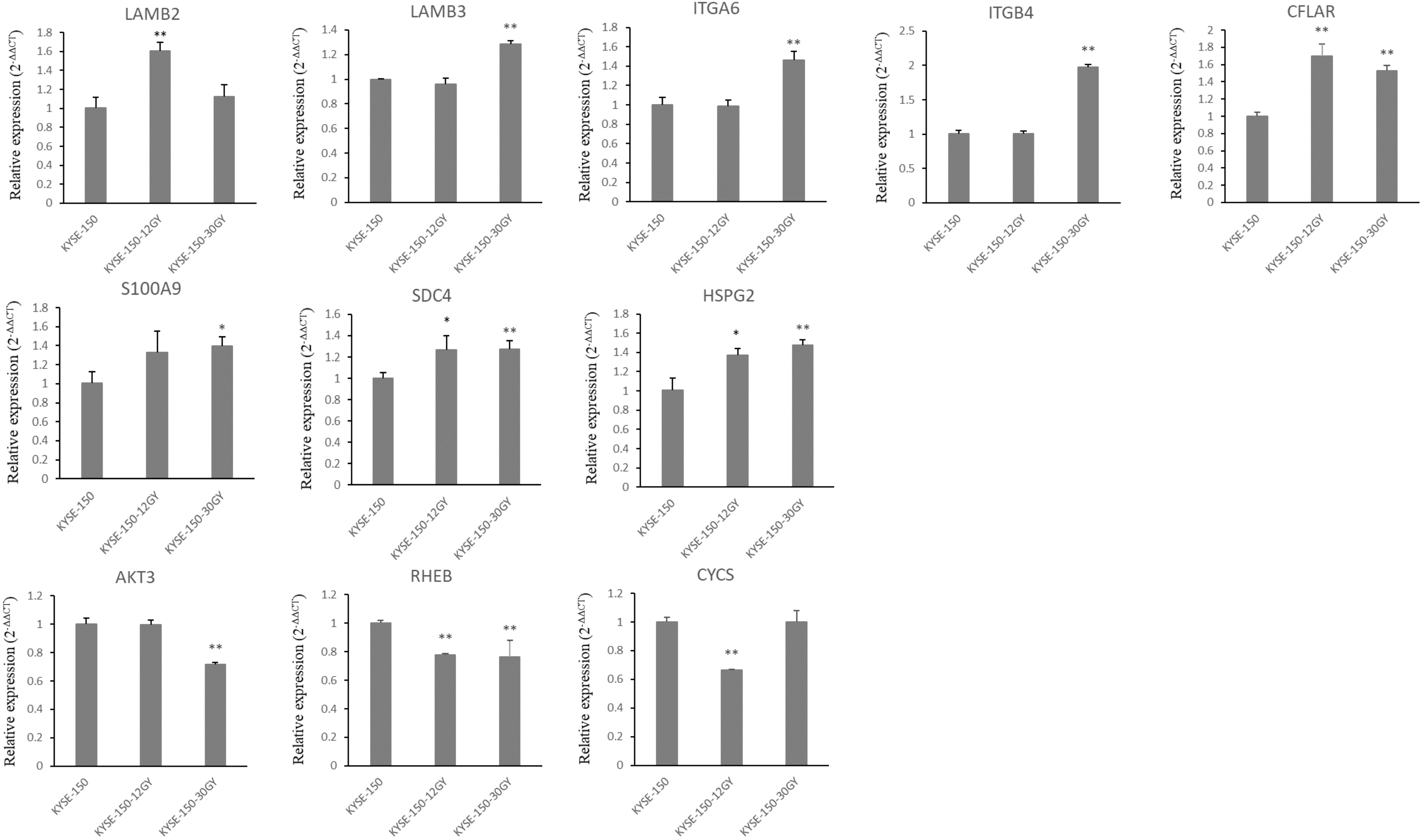

Supplementary Figure 5

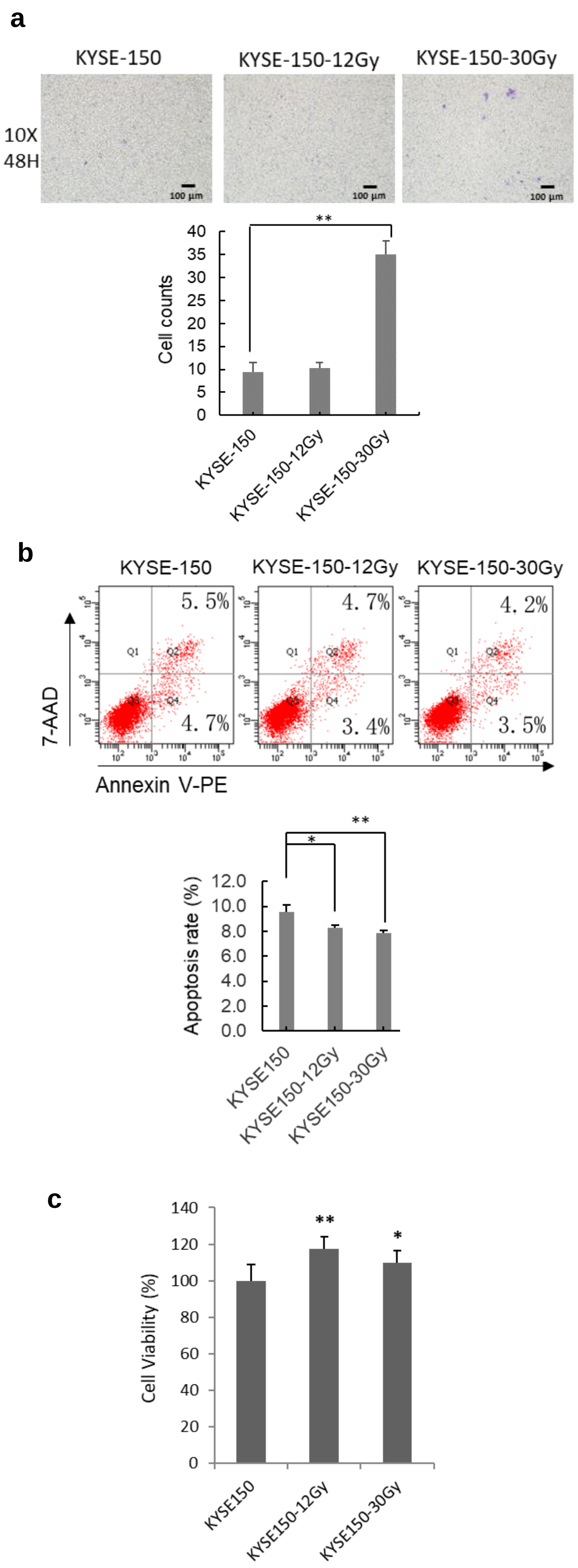

Supplement: Supplementary file 1 — Figure S1. Quality control of basic gene expression statistics. a. Replicate sequencing showed high repeatability (r = 0.96) of expression level. b. The expression distribution after depth normalization revealed similar medians for all cells analyzed. c. The expression data of discrete genes suggested a correlation of increased expression with decreased discrete degree. Therefore, we could not include genes with a normalized count ratio of less than 2 log2. d. The RSDs of gene expression assayed by duplicate sequencing appear highly linear (r = 0.98). Figure S2. Heatmap of DEGs in the three treatment groups (0 Gy controls, 12 Gy, and 30 Gy). Four gene expression patterns are seen among all DEGs. Pattern 1 shows a slight upregulation after radiation, with two subpopulations of 12 Gy cells. Pattern 2 shows a strong downregulation after 30 Gy. Pattern 3 was similar to pattern 1, but no subpopulations were observed. Finally, pattern 4 shows many slightly downregulated genes. Four cells in the 12 Gy group had a completely different expression pattern, and were excluded as outliers after sequencing depth checking. Figure S3. qPCR validation of DEGs in five radioresistant-related pathways in Fig. 4 by new treated KYSE-180 cells, KYSE-180-12 Gy cells and KYSE-180-30 Gy cells. * means P < 0.05; ** represents P < 0.01. Figure S4. qPCR validation of DEGs in five radioresistant-related pathways in Fig. 4 by KYSE-150 cells, KYSE-150-12 Gy cells and KYSE-150-30 Gy cells. * means P < 0.05; ** represents P < 0.01. Figure S5. Radioresistance-associated cellular phenotypic evidences of KYSE-150 cells. a. Transwell assay showed invasion of KYSE-150, KYSE-150-12 Gy and KYSE-150-30 Gy. b. FACS analysis with Annexin V-PE and 7-AAD showing apoptosis results of KYSE-150, KYSE-150-12 Gy and KYSE-150-30 Gy. c. Surviving KYSE-150 cells with and without FIR exposure identified by CCK-8 assay. * means P < 0.05; ** represents P < 0.01. (PDF 28921 kb) [file 12864_2019_5970_MOESM1_ESM.pdf]
